# Short-term Exposure to Particulate Matter Constituents and Mortality in a National Study of U.S. Urban Communities

**DOI:** 10.1289/ehp.1206185

**Published:** 2013-08-02

**Authors:** Jenna R. Krall, G. Brooke Anderson, Francesca Dominici, Michelle L. Bell, Roger D. Peng

**Affiliations:** 1 Department of Biostatistics, Johns Hopkins Bloomberg School of Public Health, Baltimore, Maryland, USA; 2 Department of Biostatistics, Harvard School of Public Health, Boston, Massachusetts, USA; 3 Yale School of Forestry and Environmental Studies, Yale University, New Haven, Connecticut, USA

## Abstract

Background: Although the association between PM_2.5_ mass and mortality has been extensively studied, few national-level analyses have estimated mortality effects of PM_2.5_ chemical constituents. Epidemiologic studies have reported that estimated effects of PM_2.5_ on mortality vary spatially and seasonally. We hypothesized that associations between PM_2.5_ constituents and mortality would not vary spatially or seasonally if variation in chemical composition contributes to variation in estimated PM_2.5_ mortality effects.

Objectives: We aimed to provide the first national, season-specific, and region-specific associations between mortality and PM_2.5_ constituents.

Methods: We estimated short-term associations between nonaccidental mortality and PM_2.5_ constituents across 72 urban U.S. communities from 2000 to 2005. Using U.S. Environmental Protection Agency (EPA) Chemical Speciation Network data, we analyzed seven constituents that together compose 79–85% of PM_2.5_ mass: organic carbon matter (OCM), elemental carbon (EC), silicon, sodium ion, nitrate, ammonium, and sulfate. We applied Poisson time-series regression models, controlling for time and weather, to estimate mortality effects.

Results: Interquartile range increases in OCM, EC, silicon, and sodium ion were associated with estimated increases in mortality of 0.39% [95% posterior interval (PI): 0.08, 0.70%], 0.22% (95% PI: 0.00, 0.44), 0.17% (95% PI: 0.03, 0.30), and 0.16% (95% PI: 0.00, 0.32), respectively, based on single-pollutant models. We did not find evidence that associations between mortality and PM_2.5_ or PM_2.5_ constituents differed by season or region.

Conclusions: Our findings indicate that some constituents of PM_2.5_ may be more toxic than others and, therefore, regulating PM total mass alone may not be sufficient to protect human health.

Citation: Krall JR, Anderson GB, Dominici F, Bell ML, Peng RD. 2013. Short-term exposure to particulate matter constituents and mortality in a national study of U.S. urban communities. Environ Health Perspect 121:1148–1153; http://dx.doi.org/10.1289/ehp.1206185

## Introduction

Particulate matter (PM) air pollution has been associated with a range of adverse health outcomes including mortality, hospital admissions, and lung cancer (Dominici et al. 2006; Mar et al. 2000; Ostro et al. 2006; Pope et al. 2002; Zanobetti and Schwartz 2009). PM with an aerodynamic diameter of ≤ 2.5 μm (PM_2.5_) represents a more toxic fraction of PM than other size fractions and has been consistently implicated in many health effects analyses (Burnett et al. 2000; Cifuentes et al. 2000; Peng et al. 2008; Zanobetti and Schwartz 2009). To date, most epidemiologic studies of PM have related adverse health outcomes to PM measured by mass (e.g., PM_2.5_). However, PM_2.5_ is a complex mixture of ≥ 50 chemical constituents (Bell et al. 2007), and there is increasing evidence that the chemical constituents of PM_2.5_ differ with regard to their effects on human health.

Studies of nonfatal health outcomes, including hospitalizations and birth outcomes, have suggested that health effects vary among individual PM_2.5_ constituents. A national study of cardiovascular and respiratory hospitalizations reported significant associations with elemental carbon (EC) and organic carbon matter (OCM), but not with other chemical constituents (Peng et al. 2009). Regional studies have reported positive associations of sulfate with preterm birth (Darrow et al. 2009); of EC, organic carbon, sulfate, silicon, and nitrate with emergency department visits and hospital admissions (Ito et al. 2011; Kim et al. 2012; Tolbert et al. 2007); and of EC and silicon with low birthweight (Bell et al. 2010). Zanobetti et al. (2009) reported that some constituents, including organic carbon, sodium ion, and sulfate, modified the association between short-term exposure to PM_2.5_ and hospitalizations in a study of 26 U.S. communities.

Mortality risks of different PM_2.5_ constituents have yet to be examined comprehensively at the national level. Two national-level studies reported that the PM_2.5_–mortality association differed depending on the chemical makeup of PM_2.5,_ but neither study estimated constituent-specific associations with mortality (Bell et al. 2009; Franklin et al. 2008). Local and regional time-series studies have reported estimated effects for PM_2.5_ constituents on mortality, including studies of populations in Detroit, Michigan, Seattle, Washington (Zhou et al. 2011), New York City, New York (Ito et al. 2011), and California (Ostro et al. 2007) and cities outside the United States (Cakmak et al. 2009; Cao et al. 2012). Although these studies all estimated associations between mortality and individual PM_2.5_ constituents, the specific constituents that were associated with mortality varied among the studies (e.g., organic carbon (Cakmak et al. 2009; Cao et al. 2012; Ito et al. 2011), EC (Cakmak et al. 2009; Cao et al. 2012; Ito et al. 2011; Ostro et al. 2007; Zhou et al. 2011), silicon (Ito et al. 2011; Zhou et al. 2011), sulfate (Cao et al. 2012; Ito et al. 2011), nitrate (Cao et al. 2012; Ostro et al. 2007), ammonium (Cao et al. 2012). Thus, there is uncertainty about the contributions of specific PM_2.5_ constituents to PM_2.5_-related mortality.

A national study of mortality and PM_2.5_ constituents could provide important information about the toxicity of PM_2.5_ and contribute to the scientific evidence base required to develop more targeted regulation of ambient PM. Different chemical constituents of PM_2.5_ are generated by different pollutant sources. For example, EC and OCM are often generated by motor vehicles, whereas sodium ion is associated with aerosolized sea salt (Ito et al. 2004; Schlesinger 2007; Thurston et al. 2011), although these pollutants all have multiple sources. By identifying PM_2.5_ constituents that are more toxic, we can move toward developing source-specific air pollution regulation that may be more effective at protecting public health.

Previous studies have reported regional and seasonal variation in estimated short-term health effects of different size distributions of PM, including PM_2.5_ (Bell et al. 2008; Peng et al. 2005; Zanobetti and Schwartz 2009). Because the chemical composition of PM_2.5_ varies spatially and seasonally (Bell et al. 2007), variation in estimated health effects could be driven by regional or seasonal variation in the chemical composition of PM_2.5_. This hypothesis can be explored by estimating whether associations between PM_2.5_ constituents and mortality vary seasonally or spatially. Alternatively, observed variation in estimated PM_2.5_ health effects may result from seasonal or regional differences in human activity patterns, meteorological conditions, penetration of PM_2.5_ indoors, PM_2.5_ sources, or other confounders (Peng et al. 2005).

For the present study, we estimated effects of seven major chemical constituents of PM_2.5_ on mortality: OCM, EC, silicon, sodium ion, nitrate, ammonium, and sulfate. National-level mortality effect estimates can help resolve inconsistencies in regional findings for different PM constituents. We also estimated short-term mortality effects of constituents by season and region, effects which have not been estimated across the United States previously. To our knowledge, this is the first national-level U.S. study to report estimates of the effects of individual PM_2.5_ constituents on human mortality.

## Methods

*Mortality data*. All-cause mortality data (excluding accidental deaths) were aggregated from death certificate data obtained from the National Center for Health Statistics for 2000 to 2005 (Samet et al. 2000). The original database includes mortality data for 108 urban communities (each consisting of one county or set of adjacent counties). For the present analysis, we excluded communities that were located outside the continental United States (*n* = 2 communities) or that had no PM_2.5_ constituent monitors (*n* = 29), no days with data for all seven PM_2.5_ constituents during 2000–2005 (*n* = 4), or insufficient data for model convergence (*n* = 1), leaving 72 communities for our analysis.

*PM_2.5_ constituent and weather data*. We obtained PM_2.5_ constituent data for 2000–2005 from the U.S. Environmental Protection Agency (EPA) Chemical Speciation Network, which records concentrations of > 50 chemical constituents that contribute to PM_2.5_ mass from approximately 250 monitoring sites throughout the continental United States (Bell et al. 2007; Peng et al. 2009). For daily concentrations of PM_2.5_ mass, we used data from the U.S. EPA Air Quality System from 2000 to 2005, which included approximately 1,400 monitoring sites (Dominici et al. 2006; Peng et al. 2009). We excluded data from source-oriented monitors that may not be representative of typical population exposures.

We analyzed a subset of seven constituents previously identified as covarying with PM_2.5_ total mass and/or having the largest contribution to overall PM_2.5_ total mass: OCM, EC, silicon, sodium ion, nitrate, ammonium, and sulfate (Bell et al. 2007). Together, these constituents account for 79–85% of yearly and seasonal PM_2.5_ mass (both nationally and in the eastern and western United States). Other constituents each contribute < 1% on average to the total PM_2.5_ mass (Bell et al. 2007).

Monitors typically measure PM_2.5_ constituent concentrations every third or sixth day. Organic carbon measurements were adjusted for field blanks to estimate OCM using a standard approach such that OCM = 1.4(OC_m_ – OC_b_), where OC_m_ represents measured organic carbon, OC_b_ represents organic carbon for blank filters, and 1.4 is the adjustment factor to account for non-carbon organic matter, as described previously (Bell et al. 2007).

We estimated daily community-level pollutant exposure as the arithmetic mean of daily monitor observations within the community. For communities with a single monitor, we used pollutant concentrations recorded by that monitor.

**Figure 1:**
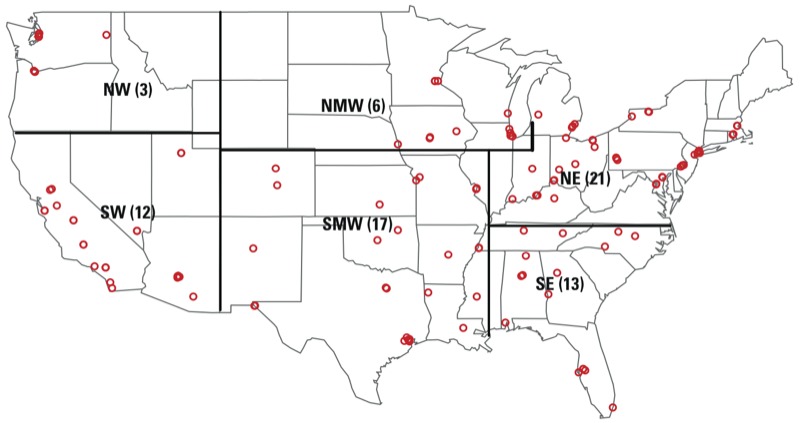
Map of the United States illustrating the 72 U.S. communities analyzed (red circles) divided into the six regions used in this analysis: NE, northeast; NMW, north midwest; NW, northwest; SE, southeast; SMW, south midwest; SW, southwest. Numbers in parentheses indicate the number of study communities within that region.

We divided the United States into six regions based loosely on U.S. EPA regions (Figure 1). Similar divisions have been used in other studies to approximately reflect variation in PM_2.5_ sources (Peng et al. 2005; Samet et al. 2000; Zanobetti and Schwartz 2009). Daily temperature and dew point temperature were obtained from the National Oceanic and Atmospheric Administration (EarthInfo 2006; Peng et al. 2009).

*Mortality risk model*. We modeled short-term associations between mortality counts and PM_2.5_ constituent concentrations with overdispersed log-linear Poisson time-series regression models. For each constituent considered, we fit a separate community-specific single-pollutant model. We chose additional covariates based on previous analyses (Peng et al. 2009; Zanobetti and Schwartz 2009). These covariates included smooth functions (natural spline) of temperature [degrees of freedom (df) = 3], 1-day lag of temperature (df = 3), and long-term and seasonal trends in mortality (df = 8/year) as well as categorical variables for age (< 65, 65–74, > 74 years) and day of week. We also estimated associations between PM_2.5_ mass and mortality.

Past research identified previous-day PM_2.5_ exposure as the exposure lag most strongly associated with mortality (Ito et al. 2011; Samet et al. 2000), and studies of PM_2.5_ constituents have corroborated this finding (Huang et al. 2012; Ito et al. 2011). We therefore included the mean value of each pollutant on the previous-day (lag 1) in single-pollutant mortality risk models. As a sensitivity analysis, we estimated mortality effects of mean exposure on the same day (lag 0) and 2 days before (lag 2). Because constituent data were not collected on consecutive days, we could not estimate effects using distributed lag models (Dominici et al. 2006).

We estimated season-specific effects by adding interaction terms between pollutant concentration and seasons to our mortality risk model. The four seasons were winter (21 December–20 March), spring (21 March–20 June), summer (21 June–20 September), and fall (21 September–20 December) (Peng et al. 2005).

To estimate national, seasonal, and regional mortality effects, we combined community-specific mortality risk estimates using a two-level normal Bayesian hierarchical model (Peng et al. 2009). To facilitate comparisons across pollutants, we report results as percent increases in mortality risk for an interquartile range (IQR) increase in pollutant concentration, with corresponding 95% Bayesian posterior intervals (95% PIs). We also report posterior probabilities that the mortality risk associated with a pollutant is greater than 0 (*p* > 0).

To analyze differences in estimated pollutant effects by season, we pooled the community-specific estimated mortality risk differences comparing each season to winter in order to obtain national-level 95% PIs for the seasonal differences. We concluded that there was no evidence of seasonal differences if these posterior intervals included zero. Because we fit separate time-series models for each community in the study, we were unable to use this same approach to explore regional differences in mortality risk. To analyze differences in risks by region, we used the pooled region-specific estimates and estimated 95% PIs for pairwise differences in mortality effect estimates between regions.

## Results

*Summary statistics*. Study communities had a combined population of 88.4 million people (2000 census) (U.S. Census Bureau 2013), with 0–254 daily nonaccidental deaths (median, 15 deaths/day). For each pollutant, the mean, minimum, and maximum days of data used in community-specific models are shown in Table 1. Although data were limited by the nondaily sampling schedule of PM_2.5_ constituent monitors, most communities (67 of 72) had ≥ 150 days of constituent data. We restricted the constituent monitor data to monitors located within the community boundaries (*n* = 141). Most communities had only one monitor collecting data (*n* = 39 communities). The other 33 communities had two monitors (*n* = 18 communities), three monitors (*n* = 9), five monitors (*n* = 2), seven monitors (*n* = 3), or eight monitors [*n* = 1 (New York City)]. Across communities, median concentrations of sulfate and OCM tended to be higher than other PM_2.5_ constituents (Table 1). Within communities, sulfate and ammonium, and OCM and EC, were highly correlated (correlation coefficients of 0.87 and 0.64, respectively); otherwise, correlations between constituent pairs were moderate or weak (Table 2).

**Table 1:** Mean (minimum–maximum) number of days of observation in the study period used for community-specific mortality risk models, IQR (median of monitor-specific IQRs), and median (minimum–­maximum) community-specific average constituent concentration (μg/m^3^).

Pollutant	No. of days	IQR	Concentration
PM_2.5_	1,636 (456–2,189)	8.00	13.6 (6.38–22.84)
OCM	388 (58–907)	3.08	4.15 (2.22–8.89)
EC	395 (58–921)	0.37	0.68 (0.29–1.51)
Silicon	395 (56–920)	0.08	0.11 (0.05–0.52)
Sodium ion	374 (58–834)	0.11	0.12 (0.04–0.60)
Nitrate	387 (58–720)	1.22	1.70 (0.50–10.05)
Ammonium	392 (58–923)	1.14	1.53 (0.34–3.90)
Sulfate	392 (58–923)	2.75	3.50 (0.71–5.91)

**Table 2:** Pairwise correlations for PM_2.5_ chemical constituents for all seasons obtained by taking the median of all monitor location-specific correlations.

	EC	Silicon	Sodium ion	Nitrate	Ammonium	Sulfate
OCM	0.64	0.20	0.10	0.22	0.47	0.42
EC	1.00	0.10	0.04	0.33	0.34	0.19
Silicon		1.00	0.09	–0.07	0.05	0.15
Sodium ion			1.00	0.12	0.04	0.10
Nitrate				1.00	0.56	0.08
Ammonium					1.00	0.87
Sulfate						1.00

**Figure 2:**
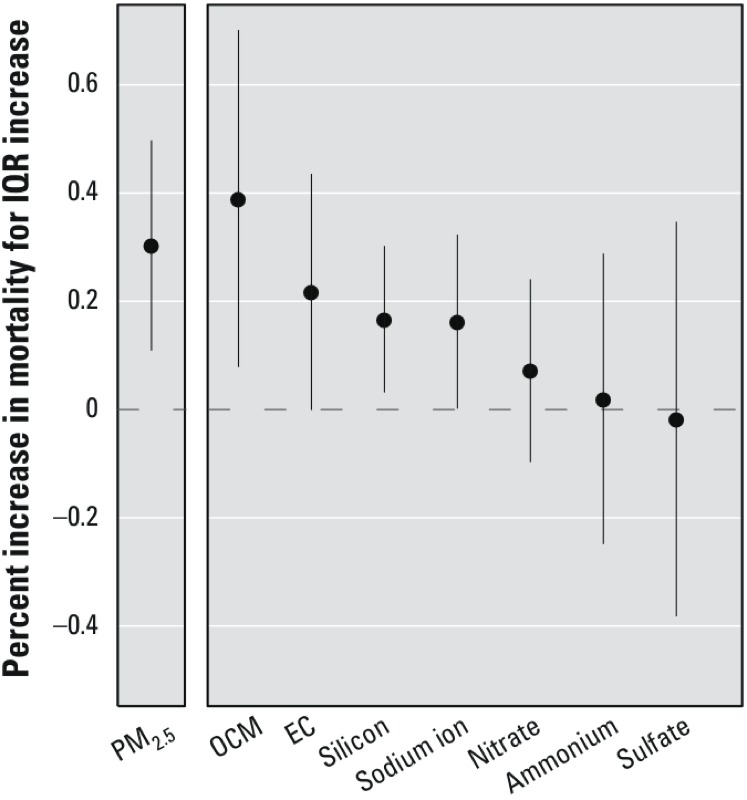
National average estimated percent increase in mortality (95% PI) associated with an IQR increase in PM_2.5_ constituents on the previous day for single-pollutant models.

*Mortality risk estimates*. We estimated that mortality increased by 0.39% (95% PI: 0.08, 0.70) in association with an IQR increase in OCM on the previous day. Mortality was also associated with IQR increases in EC (0.22%; 95% PI: 0.00, 0.44), silicon (0.17%; 95% PI: 0.03, 0.30), and sodium ion (0.16%; 95% PI: 0.00, 0.32) (Table 3, Figure 2). The posterior probability of a positive association with mortality for each of these constituents was > 0.95.

**Table 3:** National average estimated percent increase (95% PI) in mortality associated with an IQR increase in PM_2.5_ constituents on the previous day for single-pollutant and multipollutant models.

Pollutant	Single-pollutant model		Multipollutant model^*a*^	
	Estimate (95% PI)	PP (> 0)	Estimate (95% PI)	PP (> 0)
PM_2.5_	0.30 (0.11, 0.50)	1.00	—	—
OCM	0.39 (0.08, 0.70)	0.99	0.23 (–0.46, 0.92)	0.74
EC	0.22 (0.00, 0.44)	0.97	0.14 (–0.38, 0.65)	0.70
Silicon	0.17 (0.03, 0.30)	0.99	0.19 (0.00, 0.38)	0.97
Sodium ion	0.16 (0.00, 0.32)	0.98	0.10 (–0.23, 0.44)	0.72
Nitrate	0.07 (–0.10, 0.24)	0.80	—	—
Ammonium	0.02 (–0.25, 0.29)	0.56	—	—
Sulfate	–0.02 (–0.38, 0.35)	0.46	—	—
PP, posterior probability.^***a***^Explores whether the associations between mortality OCM, EC, silicon, and sodium ion in single-pollutant models are confounded by a subset of these four constituents.				

**Figure 3:**
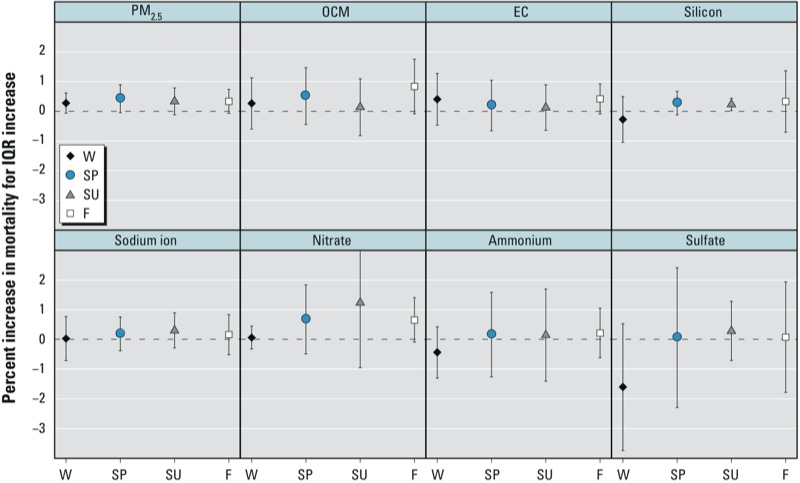
Season-specific estimated percent increase in mortality (95% PI) associated with an IQR increase in PM_2.5_ constituents on the previous day for single-pollutant models. Seasons: winter (W, 21 December–20 March), spring (SP, 21 March–20 June), summer (SU, 21 June–20 September), fall (F, 21 September–20 December).

**Figure 4:**
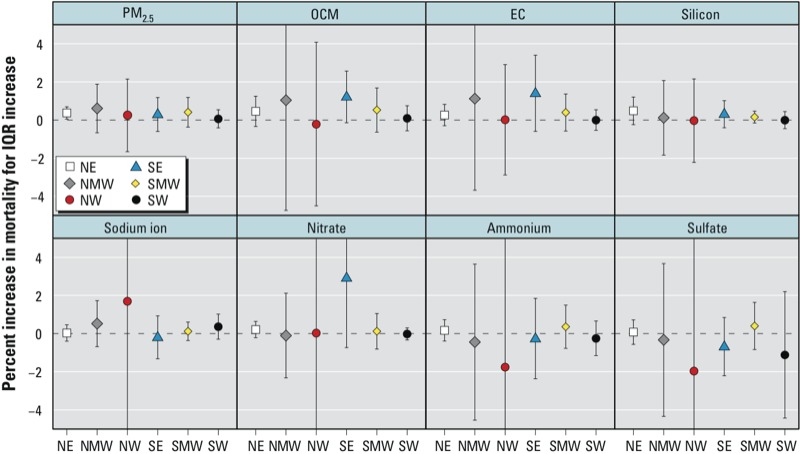
Region-specific estimated percent increase in mortality (95% PI) associated with an IQR increase in PM_2.5_ constituents on the previous day for single-pollutant models. Region designations: NE, northeast; NMW, north midwest; NW, northwest; SE, southeast; SMW, south midwest; SW, southwest.

We also estimated season-specific (Figure 3) and region-specific (Figure 4) mortality effects of PM_2.5_ constituents. We found evidence of a season-specific effect of an IQR increase in silicon on the previous day during the summer (0.23%; 95% PI: 0.03, 0.44), but no other season-specific or region-specific effect estimates were statistically significant, and we found no evidence that estimated effects of any of the seven PM_2.5_ constituents varied by season or by region using posterior intervals of the differences across seasons and regions.

An IQR increase in PM_2.5_ mass on the previous day (8.00 μg/m^3^) was associated with a 0.30% increase in mortality (95% PI: 0.11, 0.50) (Table 3, Figure 2). We found no evidence that associations between PM_2.5_ mass and mortality varied strongly by season or region, although some season-specific and region-specific associations between PM_2.5_ and mortality did rise to the level of statistical significance. For example, a 0.37% increase in mortality (95% PI: 0.05, 0.69) was associated with an IQR increase in PM_2.5_ in the northeast region (Figure 4).

As a sensitivity analysis, we estimated same-day and 2-day lagged national-average, season-specific, and region-specific mortality risks associated with PM_2.5_ and PM_2.5_ constituents, although we found little evidence of associations with mortality at these lags (see Supplemental Material, Table S1, Figures S1–S4). The national-average associations of same-day sulfate (0.29%; 95% PI: –0.10, 0.68) and ammonium (0.11%; 95% PI: –0.20, 0.42) with mortality were larger in magnitude than previous-day associations (see Supplemental Material, Table S1). We found some indication that same-day PM_2.5_ was associated with mortality nationally and in the spring and summer (see Supplemental Material, Table S1, Figure S1).

*Sensitivity analyses*. We considered several variations of our primary mortality risk model: adding a linear term for dew point temperature, increasing the degrees of freedom for both smooth functions of temperature, and including different degrees of freedom for the smooth function of time (4, 6, 10, or 12 df/year). We also tested the sensitivity of our seasonal model to season definition (winter: 1 December–28 February), and none of these alternate models produced substantially different mortality risk estimates (results not shown). When we limited data to consider cardiovascular and respiratory mortality, we found estimated effects similar to all-cause mortality (results not shown).

We fit a multipollutant mortality risk model including OCM, EC, silicon, and sodium ion simultaneously to assess whether associations found for OCM, EC, silicon, and sodium ion in single-pollutant models could be due to confounding by a subset of these four constituents (Table 3). Compared with single-pollutant model estimates, multipollutant mortality risk estimates were slightly attenuated for OCM, EC, and sodium ion and slightly increased for silicon, indicating that there was little joint confounding by the four constituent exposures. Multipollutant estimates were based on an average of 358 days of data compared with an average of 389 days for single-pollutant models. Therefore, multipollutant model estimates had larger standard errors and smaller posterior probabilities of being greater than zero than their single-pollutant counterparts.

## Discussion

We conducted a national-level study to estimate national, seasonal, and regional associations between mortality and short-term exposures to seven major constituents of PM_2.5_ mass in 72 U.S. urban communities from 2000 to 2005.

Among the seven constituents examined in this study, OCM, EC, silicon, and sodium ion were most strongly associated with mortality, with high posterior probabilities of a mortality risk larger than zero in single-pollutant models of exposure on the previous day. Epidemiologic, toxicological, and controlled human exposure studies have reported associations of EC and OCM with adverse health outcomes (Ito et al. 2011; Ostro et al. 2007; Peng et al. 2009; Rohr and Wyzga 2012; Tolbert et al. 2007). In a literature review, Rohr and Wyzga (2012) concluded that evidence supporting the toxicity of carbon-containing constituents might be stronger than for other constituents. Previous work has also indicated that silicon may be more toxic than other constituents (Franklin et al. 2008; Ito et al. 2011; Rohr and Wyzga 2012). Sodium has not been frequently implicated in previous epidemiologic and toxicological studies of PM_2.5_ constituents (Rohr and Wyzga 2012; Schlesinger 2007), although one study reported that long-term average sodium ion concentrations partially explained variability in the association between emergency admissions and PM_2.5_ across 26 communities (Zanobetti et al. 2009). Mar et al. (2006) examined sources of pollution and reported associations between sea salt, a sodium-containing source, and mortality. Some time-series studies have reported associations of adverse health outcomes with sulfate (Cao et al. 2012; Ito et al. 2011; Kim et al. 2012; Ostro et al. 2007; Zanobetti et al. 2009), nitrate (Cao et al. 2012; Ito et al. 2011; Kim et al. 2012; Ostro et al. 2007; Peng et al. 2009), and ammonium (Cao et al. 2012; Peng et al. 2009); however, studies have also found sulfate, nitrate, and ammonium to be less toxic than other constituents [e.g., sulfate (Bell et al. 2009; Peng et al. 2009; Tolbert et al. 2007), nitrate (Bell et al. 2009; Darrow et al. 2009; Franklin et al. 2008), ammonium (Bell et al. 2009; Franklin et al. 2008)].

As a sensitivity analysis, we fit a multipollutant model including OCM, EC, silicon, and sodium ion simultaneously and estimated effects that were generally similar in magnitude and direction to single-pollutant model estimates. Previous research has found multipollutant hospitalization effect estimates for EC (Levy et al. 2012) as well as for both EC and OCM (Peng et al. 2009) to be statistically significant. Our multipollutant effect estimates had large standard errors and small posterior probabilities of a positive association, so the possibility of confounding by other constituents has not been completely eliminated. On average across communities, 358 days with exposure data for all four constituents were included in multipollutant mortality risk models, and some communities had fewer days to estimate multipollutant risks compared to single-pollutant risks, which were estimated from an average of 389 days. In addition, large observed correlations between constituents (e.g., OCM/EC = 0.64) may have affected our model results.

In our analysis of PM_2.5_ total mass and mortality, we found short-term exposure to PM_2.5_ mass was associated with increased mortality, consistent with previous epidemiologic studies (Franklin et al. 2007; Ostro et al. 2006; Zanobetti and Schwartz 2009). For a 10-μg/m^3^ increase in PM_2.5_, we estimated mortality increased 0.38% (95% PI: 0.14, 0.62), whereas other national-level studies found associations of 0.74% (95% CI: 0.41, 1.07) (Franklin et al. 2008) and 0.98% (95% CI: 0.75, 1.22) (Zanobetti and Schwartz 2009). Although our point estimates were generally smaller than previously reported, methodological differences between our approach and others may explain these differences. To compare estimated PM_2.5_ mass mortality effects with estimated PM_2.5_ constituent effects, we restricted our analysis of PM_2.5_ mass to communities with data from the PM_2.5_ constituent monitoring network, which is a smaller set of communities than studies focusing on PM_2.5_ total mass have previously examined (Dominici et al. 2006; Zanobetti and Schwartz 2009).

We found little evidence of regional or seasonal variation in associations between mortality and PM_2.5_ constituents or total mass PM_2.5_. Past work has suggested seasonal trends in constituent-specific mortality effects, although results are somewhat ambiguous across studies. Constituent-mortality associations were larger in magnitude during the cooler part of the year than during warmer months in California and in a Chinese city (Huang et al. 2012; Ostro et al. 2007), whereas a study in New York City reported significant associations of PM_2.5_ constituents with mortality in the warm season but not the cold season (Ito et al. 2011). Silicon and EC were more associated with mortality in the cold season in Seattle, but constituent-mortality associations were similar between seasons in Detroit (Zhou et al. 2011).

In general, the power to detect seasonal and regional differences in PM_2.5_ mass and PM_2.5_ constituent mortality effects in the present study was limited because of the infrequent measurement of the constituent exposures, the relatively short time series, and the small number of ambient monitor locations, particularly in the western United States. Unlike previous studies, we did not find evidence that PM_2.5_ mass mortality effect estimates varied spatially or seasonally (Dominici et al. 2006; Franklin et al. 2007, 2008; Zanobetti and Schwartz 2009). Model differences may partially explain this discrepancy because earlier seasonal studies used the mean concentration at lags 0 and 1 on season-stratified data (Zanobetti and Schwartz 2009). In addition, we explicitly tested for seasonal and regional differences using posterior intervals. Peng et al. (2005) documented seasonal and regional variations in estimated effects of PM on mortality, but these estimates were for exposure to PM_10_ (≤ 10 μm in aerodynamic diameter) during an earlier time period (1987–2000). The seasonal and regional differences previously reported may be difficult to observe using more recent data because of declining associations between PM and mortality (Dominici et al. 2007). If seasonal and regional differences in PM_2.5_ mortality effects are explained by differences in the chemical composition of PM_2.5_, we would not expect to find seasonal or regional differences in associations between PM_2.5_ constituents and mortality, which is consistent with our findings. However, in contrast with previous studies, we also did not find evidence of regional or seasonal variation in associations between PM_2.5_ and mortality; consequently, our analysis does not clarify whether previously observed differences in estimated effects of PM_2.5_ on mortality were driven by differences in chemical composition.

*Limitations*. We focused on seven constituents that make up the largest fraction of PM_2.5_. However, if PM_2.5_ mass has an effect on mortality that is not mediated through its chemical composition, then we might be more likely to spuriously identify constituents as harmful because they are correlated with PM_2.5_ mass. Future work could apply different regression techniques to distinguish among associations attributable to chemical composition versus PM_2.5_ mass (Mostofsky et al. 2012). In addition, the seven constituents that we evaluated may be correlated with toxic constituents that contribute less to PM_2.5_ by mass. For example, Ito et al. (2004) identified an oil source of PM in New York City that contained nitrate as well as nickel and vanadium, constituents that contribute less to PM_2.5_ by mass, but may be more toxic than more major constituents (Bell et al. 2010; Franklin et al. 2008). However, constituents such as nickel and vanadium often have large proportions of daily data below monitor detection limits (Burnett et al. 2000) and, therefore, may pose additional challenges to analysis. Associations with a given PM_2.5_ chemical component should be considered as potentially indicative of associations with another component or set of components with similar sources.

In our health effects analysis, we did not account for exposure misclassification, which has been demonstrated in previous work (Bell et al. 2011). Depending on the type of measurement error, estimated health effects of estimated community-level exposures may be biased (Zeger et al. 2000). We did not address error resulting from the use of ambient exposure data rather than personal exposure data, which are not available on the national scale or for long time frames (Dominici et al. 2000). However, a simulation study suggested that improved exposure prediction may not always improve health effect estimation (Szpiro et al. 2011). Using population-weighted community-level exposure data also may not substantially change estimated relative risks (Chang et al. 2011).

Although we performed a sensitivity analysis using different time periods to define seasons, we could not model a smooth transition in the magnitude of associations between pollutants and mortality between consecutive seasons. Further, potential confounders for each season (e.g., weather) may differ by location and may require community-specific modeling approaches. Our approach was to use the same model for each community, and further work may be needed to explore the sensitivity of season-specific estimates to modeling of confounders that vary by location.

Most air pollution health effects studies estimate community-level ambient average pollutant concentrations using the arithmetic mean of monitor concentrations, as we did (Ostro et al. 2007; Peng et al. 2009; Samet et al. 2000). A previous simulation study suggested that health effect estimates were less biased when the community-level ambient average was estimated using a spatial model rather than the simple arithmetic mean of data from monitors in each community, as we did for the present study (Peng and Bell 2010). Future work could incorporate spatial modeling to estimate community-level pollutant exposure (Choi et al. 2009). Although distributed lag models are preferred when estimating the effect of pollution over multiple days of exposure (Dominici et al. 2006; Zanobetti and Schwartz 2009), we could not fit distributed lag models using our non-daily PM_2.5_ constituent data.

## Conclusions

Our analysis substantially builds upon previous studies of PM_2.5_ constituents by providing the first comprehensive national-level assessment of associations between nonaccidental mortality and seven PM_2.5_ constituents in 72 urban communities across the United States during 2000–2005. We found evidence of associations between mortality and OCM, EC, silicon, and sodium ion. We did not find evidence that chemical constituent mortality risks varied by season or region. However, we also did not find evidence of seasonal or regional variation in associations between PM_2.5_ and mortality, in contrast with previous studies. Our study found evidence that some chemical constituents of PM_2.5_ were more associated with mortality than others, which may indicate that regulating PM solely by mass will not sufficiently protect human health.

## Supplemental Material

(283 KB) PDF
