# Supplementary material for: Short-term Exposure to Particulate Matter Constituents and Mortality in a National Study of U.S. Urban Communities
Source: Environ Health Perspect. 2013 Aug 2;121(10):1148–53. doi: 10.1289/ehp.1206185 (PMC3801200; doi:10.1289/ehp.1206185)

Supplemental Material

Short-term Exposure to Particulate Matter Constituents and Mortality in a National Study of U.S. Urban Communities

Jenna R. Krall, G. Brooke Anderson, Francesca Dominici, Michelle L. Bell, Roger D. Peng

Table of Contents

Table S1: National-average mortality risk estimates for lags 0 and 2 (page 2)

Figure S1: Season-specific mortality risk estimates for lag 0 (page 3)

Figure S2: Season-specific mortality risk estimates for lag 2 (page 4)

Figure S3: Region-specific mortality risk estimates for lag 0 (page 5)

Figure S4: Region-specific mortality risk estimates for lag 2 (page 6)

Supplemental Material, Table S1: National average estimated percent increase in mortality associated with an IQR increase in $PM_{2.5}$ constituents on the same day (lag 0) and two days before (lag 2) for single pollutant models.

	Lag 0		Lag 2	
Pollutant	Estimate (95% PI[a])	P(>0)[b]	Estimate (95% PI[a])	P(>0)[b]
$PM_{2.5}$	0.15 (-0.03, 0.34)	0.95	-0.01 (-0.20, 0.18)	0.44
OCM	-0.04 (-0.38, 0.29)	0.40	0.17 (-0.13, 0.47)	0.87
EC	-0.14 (-0.38, 0.10)	0.13	0.14 (-0.08, 0.36)	0.89
Silicon	0.03 (-0.13, 0.20)	0.65	0.01 (-0.14, 0.17)	0.56
Sodium Ion	-0.01 (-0.17, 0.16)	0.46	0.00 (-0.15, 0.16)	0.51
Nitrate	-0.01 (-0.21, 0.19)	0.46	0.04 (-0.15, 0.24)	0.67
Ammonium	0.11 (-0.20, 0.42)	0.76	-0.06 (-0.38, 0.25)	0.35
Sulfate	0.29 (-0.10, 0.68)	0.93	-0.26 (-0.64, 0.12)	0.09

[a] 95% PI: 95% posterior intervals for the mortality effect estimate

[b] P(>0): posterior probabilities that the mortality effect estimate is greater than 0

Supplemental Material, Figure S1: Season-specific estimated percent increase in mortality (95% posterior intervals [95% PI]) associated with an IQR increase in $PM_{2.5}$ constituents on the same day (lag 0) for single pollutant models. Seasons are defined: winter (w: December 21 – March 20), spring (sp: March 21 – June 20), summer (su: June 21 – September 20), fall (f: September 21 – December 20).

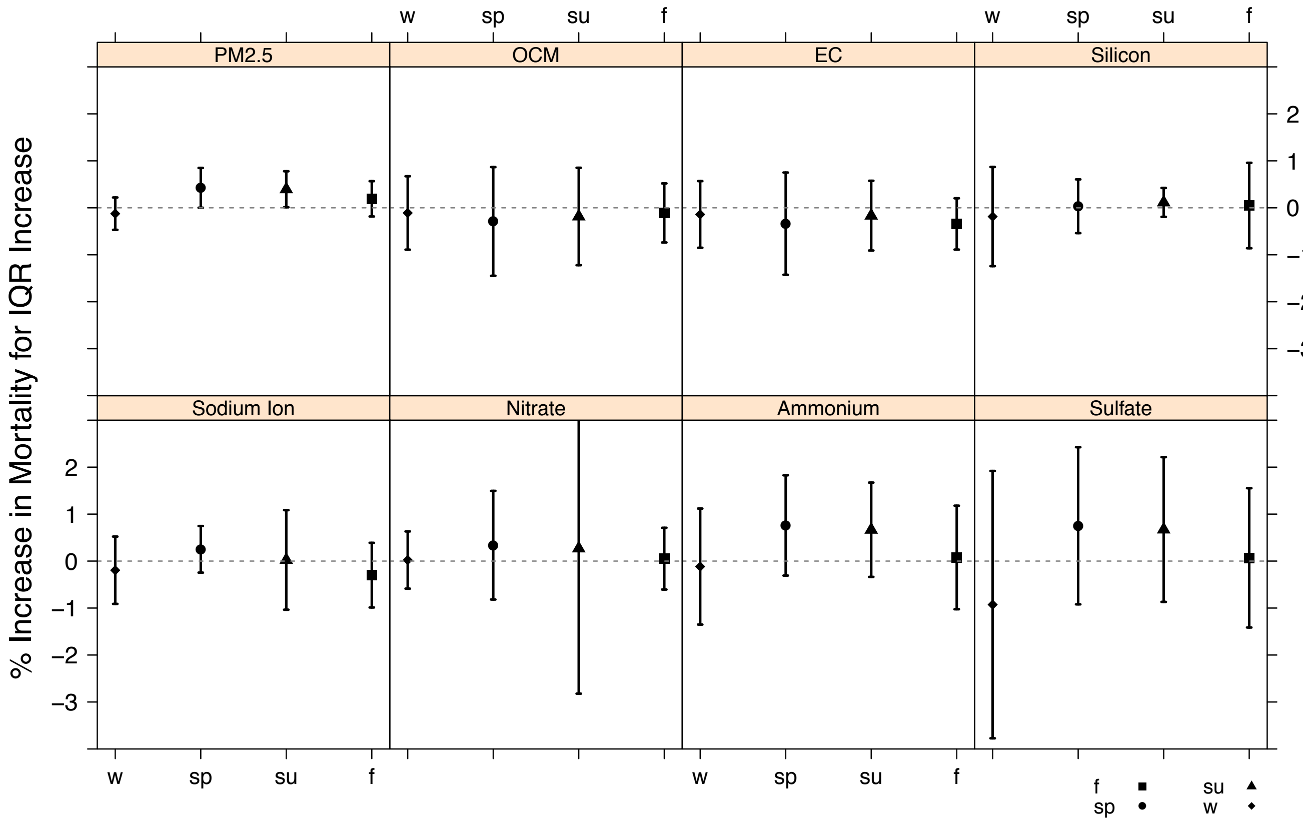

Supplemental Material, Figure S2: Season-specific estimated percent increase in mortality (95% posterior intervals [95% PI]) associated with an IQR increase in $PM_{2.5}$ constituents two days before (lag 2) for single pollutant models. Seasons are defined: winter (w: December 21 – March 20), spring (sp: March 21 – June 20), summer (su: June 21 – September 20), fall (f: September 21 – December 20).

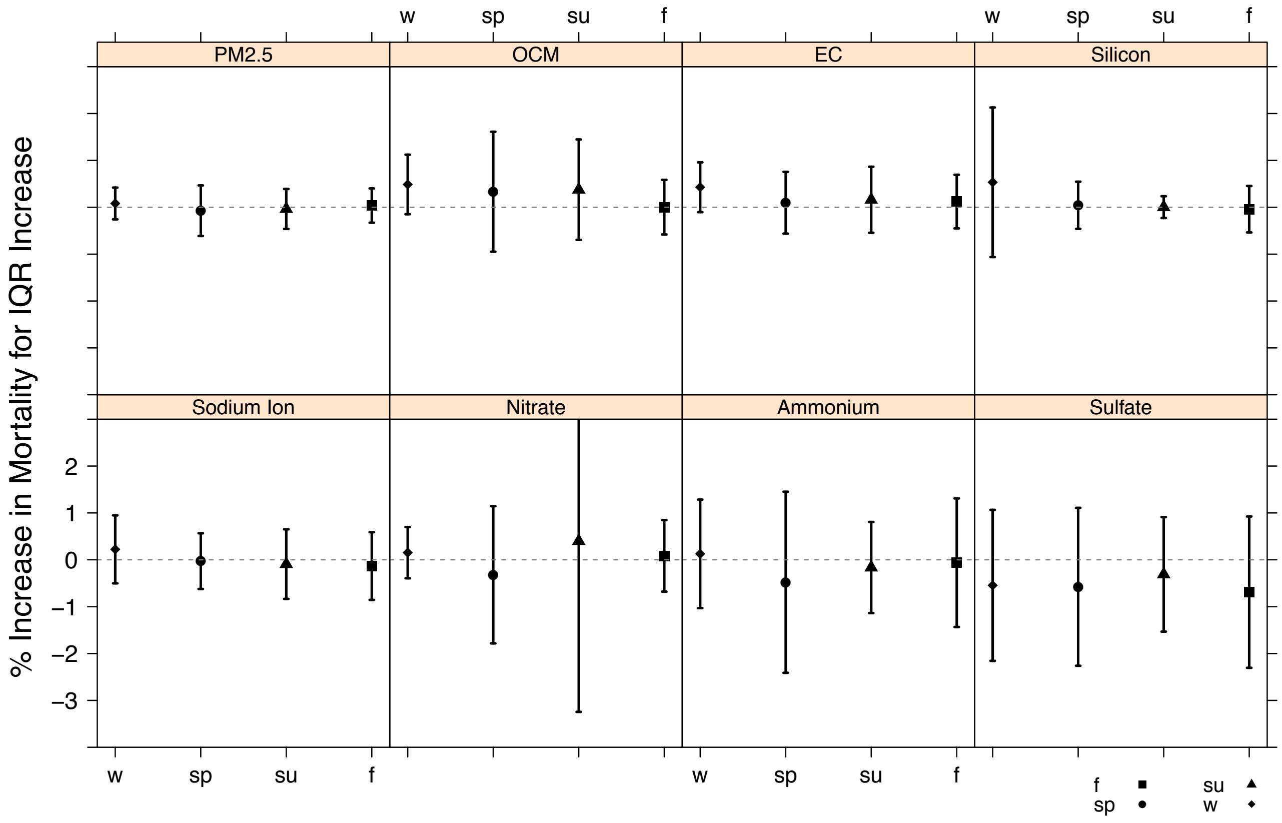

Supplemental Material, Figure S3: Region-specific estimated percent increase in mortality (95% posterior intervals [95% PI]) associated with an IQR increase in $PM_{2.5}$ constituents on the same day (lag 0) for single pollutant models. Region designations include: NE, northeast; NMW, north midwest; NW, northwest; SE, southeast; SMW south midwest; SW southwest. See Figure 1 in the main text for a map of the regions.

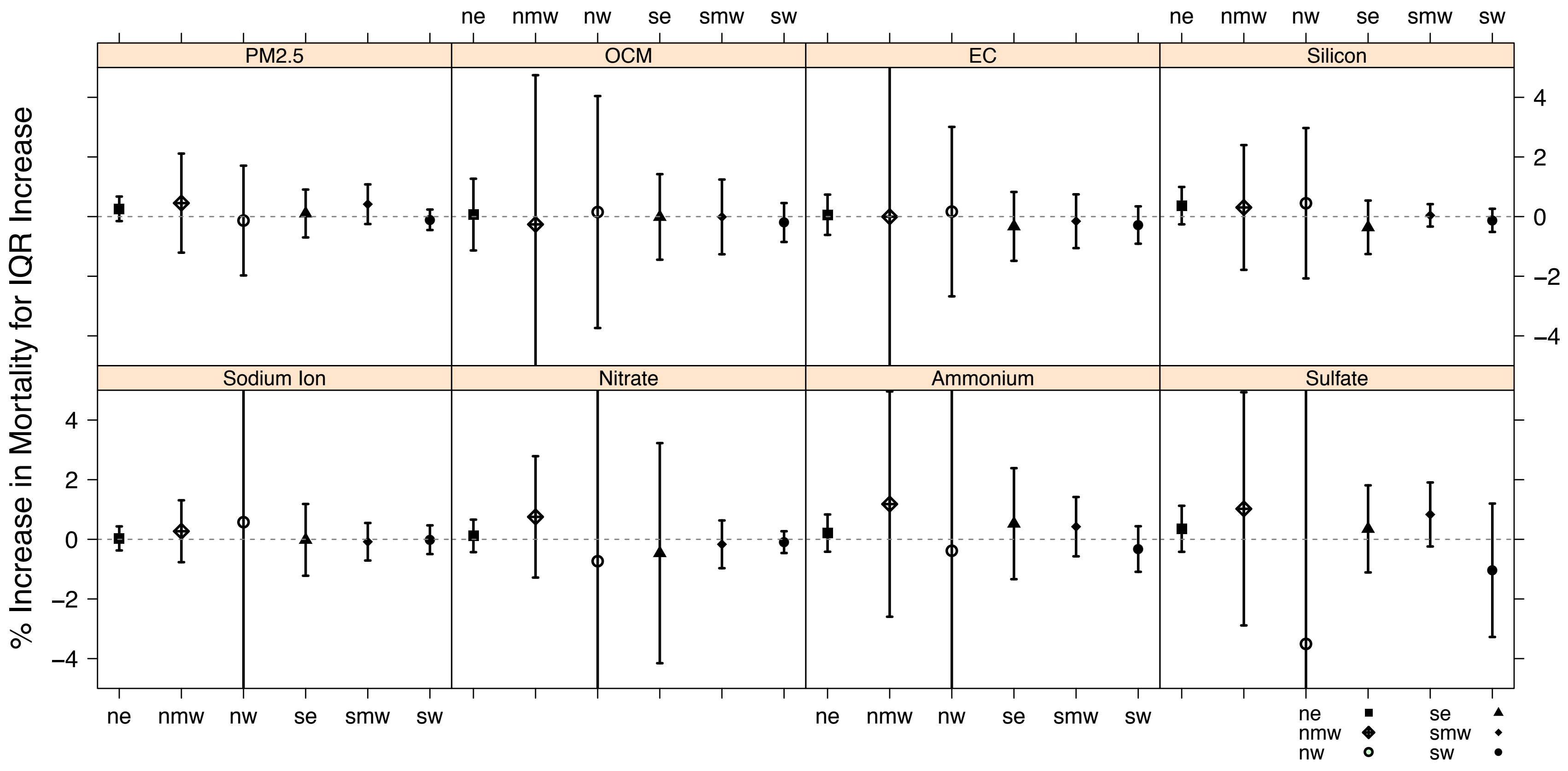

Supplemental Material, Figure S4: Region-specific estimated percent increase in mortality (95% posterior intervals [95% PI]) associated with an IQR increase in $PM_{2.5}$ constituents on two days before (lag 2) for single pollutant models. Region designations include: NE, northeast; NMW, north midwest; NW, northwest; SE, southeast; SMW south midwest; SW southwest. See Figure 1 in the main text for a map of the regions.

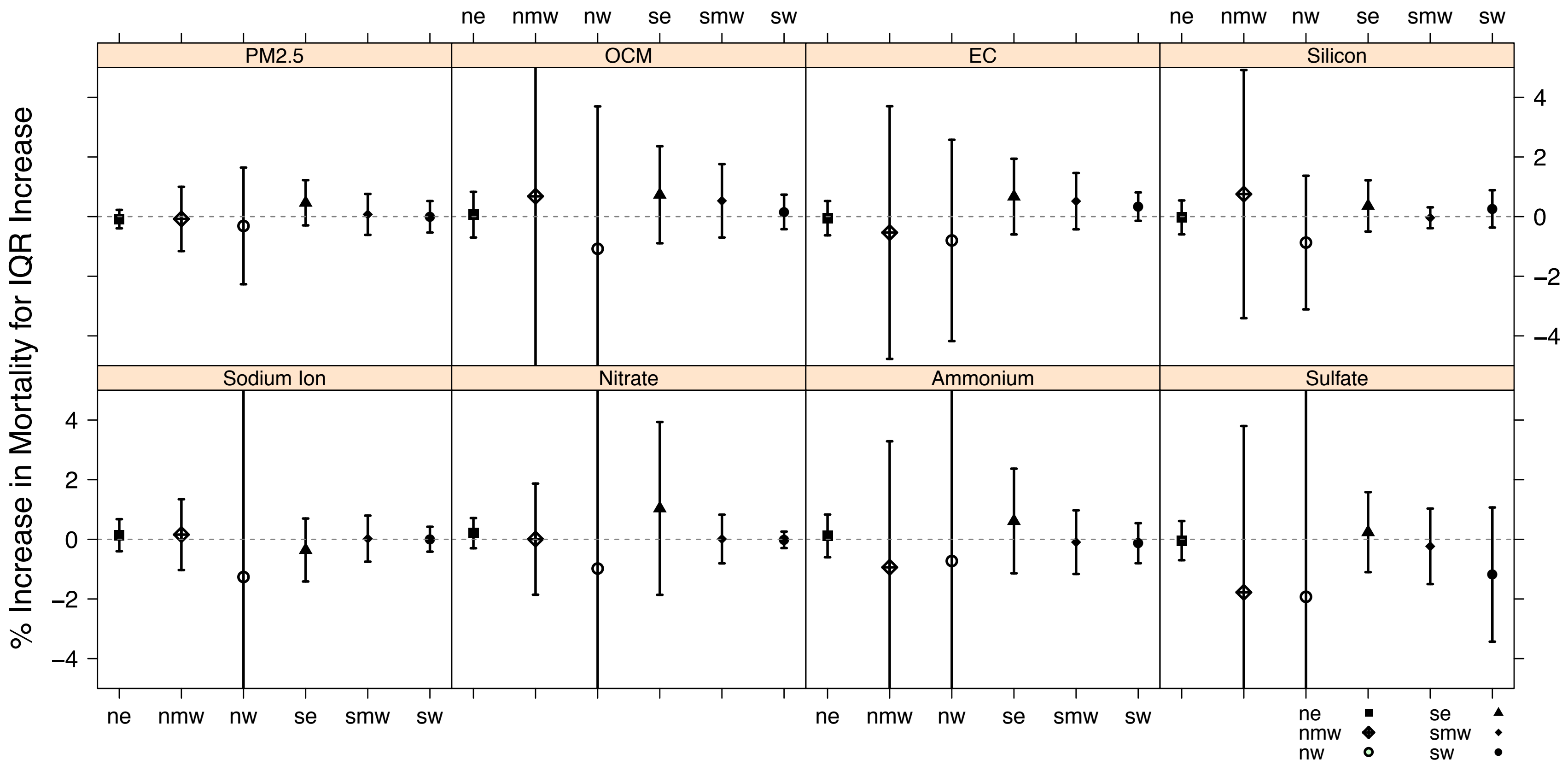

Supplement: (283 KB) PDF [file ehp.1206185.s001.pdf]
